# The Accumulation and Prognosis Value of Tumor Infiltrating IL-17 Producing Cells in Esophageal Squamous Cell Carcinoma

**DOI:** 10.1371/journal.pone.0018219

**Published:** 2011-03-31

**Authors:** Lin Lv, Ke Pan, Xiao-dong Li, Ke-lin She, Jing-jing Zhao, Wei Wang, Ju-gao Chen, Yi-bin Chen, Jing-ping Yun, Jian-chuan Xia

**Affiliations:** 1 State Key Laboratory of Oncology in Southern China and Department of Experimental Research, Cancer Center, Sun Yat-sen University, Guangzhou, People's Republic of China; 2 Department of Thoracic Oncology, Cancer Center, Sun Yat-sen University, Guangzhou, People's Republic of China; 3 Department of Pathology, Cancer Center, Sun Yat-sen University, Guangzhou, People's Republic of China; 4 Department of Gastric and Pancreatic Surgery, Cancer Center, Sun Yat-sen University, Guangzhou, People's Republic of China; University of Palermo, Italy

## Abstract

**Background:**

The role of IL-17 producing cells in tumors is controversial. In the present study, we investigated the prognostic value of measuring tumor-infiltrating IL-17 producing cell levels in human esophageal squamous cell carcinoma (ESCC).

**Methodology/Principal Findings:**

Immunohistochemical staining was performed to investigate the levels of IL-17+ tumor infiltrating lymphocytes (TILs), as well as CD8+ cytotoxic T lymphocytes (CTLs) and CD57+ natural killer (NK) cells from 181 ESCC patients. The prognostic value of measuring the densities of IL-17+TILs and the correlation with CTLs and NK was evaluated. IL-17 producing cells were detected in esophageal squamous cell carcinoma tissues. The IL-17 producing cells were major CD4 positive, but Foxp3 negative. The median level of IL-17+TILs was 3.90 cells/high power microscopic field (HPF). The density of IL-17 producing cells correlated negatively with T stage (*P* = 0.042). The higher densities of tumor infiltrating IL-17+ lymphocytes were associated with better overall survival (*P* = 0.031). Furthermore, we found that there were positive correlations between levels of IL-17 producing cells and the densities of CD8+cells, as well as CD57+cells (r = 0.198, *P* = 0.008 for CD8+ cells and r = 0.261, *P*<0.001 for CD57+ cells, respectively). The prognosis analysis also showed that the higher levels of CD8+ CTLs and CD57+ NK cells correlated with better overall survival of ESCC patients.

**Conclusions:**

Our study suggests that tumor infiltrating IL-17 producing cells in ESCC patients may have protective roles in the tumor microenvironment and may be treated as a prognostic marker for ESCC patients.

## Introduction

Substantial evidence indicates that the abundance of tumor-infiltrating lymphocytes in the microenvironment of certain tumor types is associated with the prognosis of cancer patients. Moreover, each subset of tumor-infiltrating lymphocytes has a unique role in the antitumor response [1]–[4]. The presence of tumor-infiltrating cytotoxic T lymphocytes (CTLs) and natural killer (NK) cells correlates with improved survival and confers antitumor activity [5]. However, other tumor-infiltrating lymphocyte subsets exhibit bipolar roles: promoting tumor growth or inhibiting tumor progression [6]. These subsets include the newly identified tumor-infiltrating IL-17 producing cells. Interleukin-17 (IL-17), originally termed CTLA-8, plays an important role in inflammation and autoimmune diseases in both mice and humans [7]–[13]. Early research focused on the roles and mechanisms of IL-17 producing cells in inflammation and autoimmune diseases. Because chronic inflammation were correlated significantly to tumor invasion, migration and metastasis [14], [15], scientists have begun to pay more attention to the significance of IL-17 in tumor models. There is accumulating evidence that IL-17 producing cells are present in various cancers, including ovarian cancer, breast cancer, non-small cell lung cancer, hepatocellular carcinoma and gastric cancer [16]–[19]. Substantial evidence indicated that IL-17 was produced mainly by CD4+ T lymphocytes, and these cells were defined as T helper 17 (Th 17) cells [14], [15], [20]. However, in recent studies, it was found that other T cell subsets can also produce IL-17, such as NKT, gamma-delta T cells and Tregs, including mouse models and human beings [14], [20]–[26]. Although IL-17 producing cells have been detected in various tumors, their effect on tumor cell survival and exact physiological role in tumor immunity remain controversial. IL-17 producing cells could enhance tumor growth by promoting angiogenesis [14], [18]. Conversely, IL-17 producing cells might promote tumor regression by enhancing antitumor immunity [15], [27]–[30].

Esophageal squamous cell carcinoma (ESCC) is the major histological type of esophageal cancer in the "Esophageal Cancer Belt," which stretches westward from China through central Asia to northern Iran [31], [32]. ESCC is the eighth most common cancer worldwide [33], and ranks the sixth cancer mortality worldwide [34]. It was reported that the host immune response prompted by ESCC may influence patient prognosis; both adaptive and innate immunity play important roles in ESCC progression and regression [35]–[38]. Yasushi et al showed that the number of CD8+ T cells correlated with favorable outcomes in ESCC patients [39]. Hsia et al found that ESCC patient prognosis correlated positively with intratumoral NK cell infiltration [36]. Xue et al found that FOXP3 expression was associated with lymph node metastasis and pathological TNM staging, suggesting that regulatory T cells (Tregs) might promote tumor progression [38]. However, up until now, the presence and clinical significance of IL-17 producing cells have not been previously studied in ESCC. Thus, in this study, we evaluated the accumulation and clinicopathological significance of tumor-infiltrating IL-17 producing cells in tumor tissues from ESCC patients. The prognosis value of IL-17 producing cells was also evaluated. Furthermore, we detected CD8+ CTLs and CD57+ NK cells in the same tumor tissues and relationships between the number of IL-17 producing cells and the density of CD8+ CTLs or CD57+ NK cells were further evaluated.

## Results

### Immunohistochemical staining of IL-17 producing cells and their associations with clinicopathological characteristics

**Figure 1:**
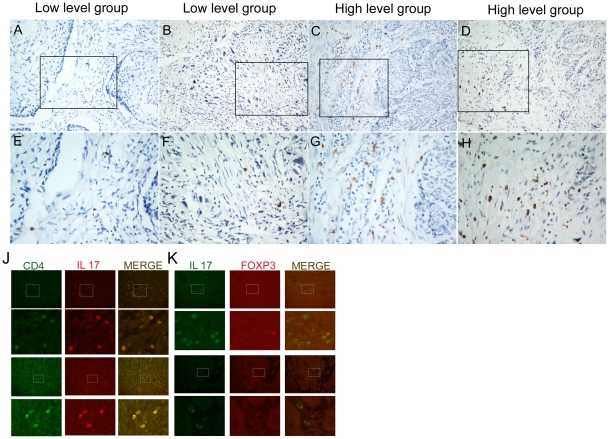
Representative immunohistochemical staining photomicrographs of IL-17 producing cells in tumor tissues of ESCC. Positive staining of IL-17 was detected in cellular cytoplasm of tumor tissues. (A), (B), (E) and (F): Low density of IL-17+ TILs. (C), (D), (G) and (H): High density of IL-17+ TILs. (J) and (K): Double stainings of CD4 (green, on the menbrane) and IL-17 (red, in the cytoplasm), IL-17 (green, in the cytoplasm) and FOXP3 (red, nuclei) in paraffin-embedded specimens were analyzed by immunofiuorescence. Original magnification: A–D×200; E–K×400.

The representative photomicrographs of tissue sections immunostained for IL-17 are shown in Figure 1. IL-17 producing cells were detected in esophageal squamous cell carcinoma tissue. In order to clarify which set of T cells could produce IL-17, double immunohistochemical staining was performed in the same tissue section. Immunofiuorescence results showed that IL-17 producing cells were major CD4 positive, but FOXP3 negative in ESCC tissues (Figure 1). The median value of IL-17 producing cells was 3.90 cells/HPF (range: 0.00–21.40 cells/HPF).

The associations between the levels of IL-17 producing cells and clinicopathological factors of the ESCC patients are summarized in Table 1. According to previous studies, patients were divided into two groups based on the median of IL-17 producing cells (high level group vs. low level group) [4], [18]. There was a significant inverse correlation between the densities of IL-17+ TILs and T (depth of primary tumor invasion, *P* = 0.042).

**Table 1:** Relationship between IL-17 producing cell density and clinical-pathologic factors.

Variables	Number of Patients	IL-17 producing cell density		*P* value
		Low level group, n = 90	High level group, n = 91	
**Age(years)**				0.506
<60	105	50	55	
≥60	76	40	36	
**Gender**				0.080
Male	141	75	66	
Female	40	15	25	
**Tumor length (cm)**				0.110
<5	75	32	43	
≥5	106	58	48	
**Differentiation**				0.199
G1	45	26	19	
G2	85	39	46	
G3	51	25	26	
**Location**				0.952
Upper third	13	6	7	
Middle third	113	56	57	
Lower third	55	28	27	
**T**				0.042*
T1+T2	57	22	35	
T3+T4	124	68	56	
**N**				0.335
No	101	47	54	
Yes	80	43	37	
**M**				0.444
M0	175	86	89	
M1	6	4	2	
**TNM staging**				0.177
Stage I-II	117	53	64	
Stage III-IV	64	37	27	

G1, well differentiated; G2, moderately differentiated; G3, poorly differentiated; T, depth of primary tumor invasion; N, regional lymph nodes; M, distant metastasis; TNM, tumor-lymph node-metastasis classification.

* Statistically significant (*P*<0.05).

### Correlation between the densities of IL-17 producing cells and patients' survival

**Figure 2:**
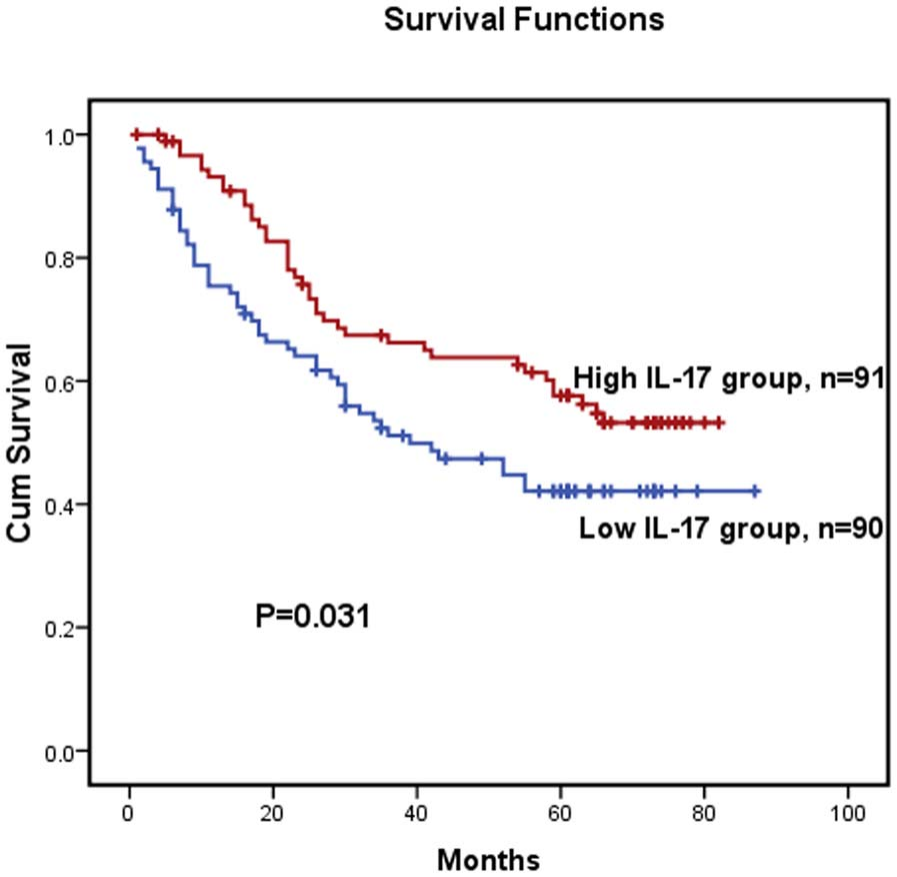
Kaplan–Meier survival curves of ESCC patients (n = 181) after surgical resection. Increased IL-17-producing cells correlate with improved patient survival. Patients in high IL-17-producing cell density group exhibited significantly better survival than the low IL-17-producing cell density group (log-rank test: *P* = 0.031).

The median survival time of the 181 ESCC patients was 44 months (range 1–87 months). The five-year survival rate was 49.9%. The overall survival curves of the patients in this study are depicted in Figure 2. The statistical analysis demonstrated a positive correlation between overall survival and the density of IL-17+ TILs (Figure 2, long-rank test: *P* = 0.031).

The univariate analysis demonstrated that IL-17 producing cell density (*P* = 0.006), differentiation (*P* = 0.006), T (depth of tumor invasion, *P* = 0.002), N (lymph node metastases, *P*<0.001) and TNM staging (*P*<0.001) were significantly associated with overall survival (Table 2). The subsequent multivariate analysis, however, indicated that only differentiation was an independent predictor for overall survival in ESCC (*P* = 0.030, Table 2).

**Table 2:** Univariate and multivariate analyses of variables associated with overall Survival.

Variables	Univariate analysis			Multivariate analysis		
	HR	95% CI	*P* value	HR	95% CI	*P* value
IL-17+TIL (high vs. low)	0.633	0.416–0.964	0.033*	0.662	0.431–1.015	0.058
Age, (≥60 vs. <60)	1.141	0.752–1.731	0.535			
Gender (female vs. male)	0.813	0.490–1.351	0.425			
Location(lower/middle/upper)	1.093	0.754–1.585	0.638			
Length, (≥5 vs. <5)	1.226	0.801–1.876	0.348			
Differentiation (G3/G2/G1)	1.510	1.123–2.032	0.006*	1.384	1.033–1.856	0.030*
T (T3+T4 vs. T1+T2)	2.233	1.330–3.750	0.002	1.605	0.875–2.942	0.126
N (Yes vs. No)	3.235	2.103–4.979	<0.001*	2.176	1.001–4.728	0.050
M (M1 vs. M0)	2.328	0.944–5.741	0.067			
TNM (III+IV vs. I+II)	3.477	2.282–5.298	<0.001*	1.433	0.632–3.251	0.389

* Statistically significant (*P*<0.05).

### Relationship between the levels of IL-17+ TILs and CD8+ CTLs cells as well as CD57+ NK cells in tumor microenvironment

**Figure 3:**
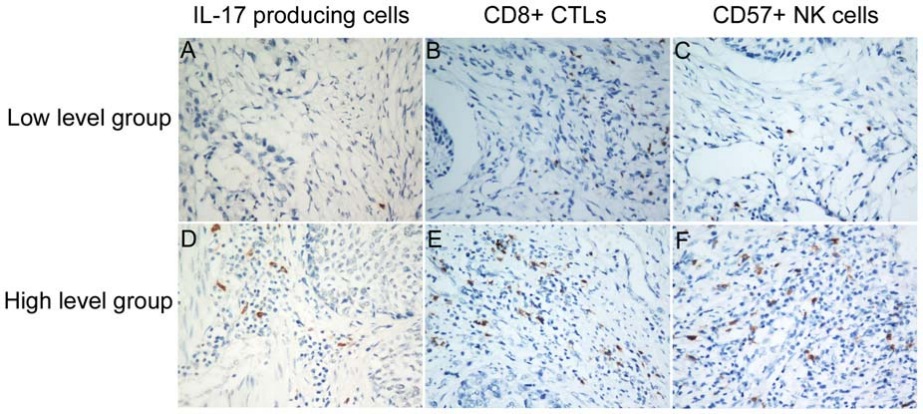
Representative photomicrographs showing immunohistochemical staining of IL-17, CD8 and CD57 in the same ESCC tissues. (A) Low density of IL-17 producing cells. (B) Low density of CD8+ CTLs cells. (C) Low density of CD57+ NK cells. (D) High density of IL-17 producing cells. (E) High density of CD8+ CTLs cells. (F) High density of CD57+ NK cells (original magnification ×400).

**Figure 4:**
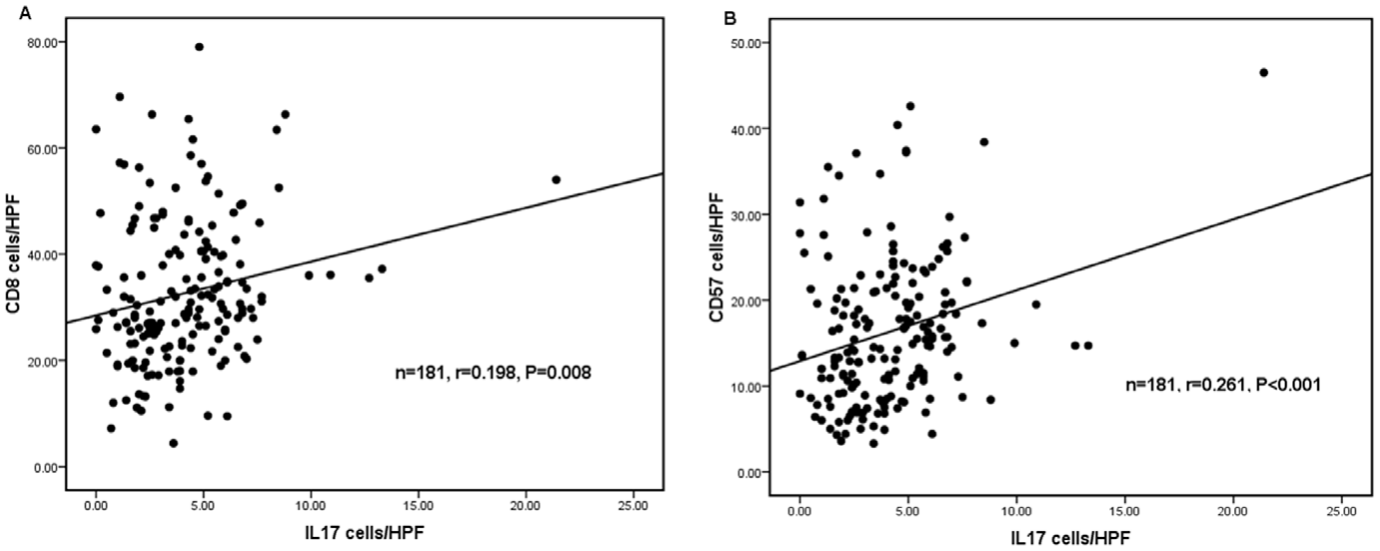
IL-17 producing cells correlated positively with the densities of CD8+ CTLs and CD57+ NK cells. The correlation between the densities of IL-17 producing cells and CD8+T lymphocytes (A), CD57+NK cells (B). The samples were divided into two groups based on the median value of IL-17 producing cells.

**Figure 5:**
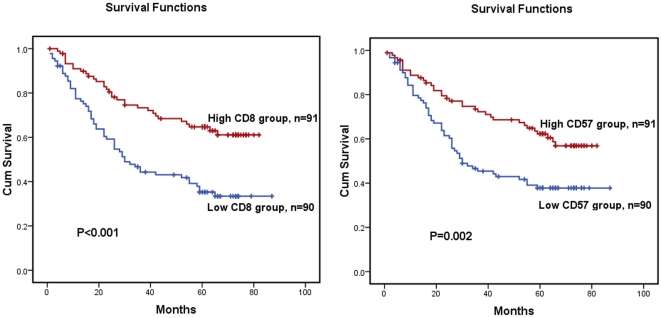
Kaplan–Meier survival curves of ESCC patients (n = 181) after surgical resection. Increased tumor-infiltrating CD8+ CTLs and CD57+ NK cells predict improved patient survival. (A) The survival rate for patients in the high CD8+ CTLs density group was significantly better than that for patients in the low density group (log rank test, *P*<0.001). (B) Kaplan–Meier survival curves for high CD57+ NK cell density group versus low CD57+ NK cell density group showed a highly significant separation (log-rank test: *P* = 0.002).

The levels of CD8+ CTLs and CD57+ NK cells in tumor tissue in ESCC patients were evaluated. In addition, the Pearson correlation coefficient was calculated, and linear regression analysis was applied to assess the relationships between the IL-17 producing cells and CD8+ CTLs or CD57+ NK cells in the tumor microenvironment. Infiltrating CD8+ CTLs and CD57+ NK cells were detected in tumor tissues by immunohistochemical staining (Figure 3), and statistical analysis showed that the levels of IL-17 producing cells positively correlated with levels of CD8+ CTLs in tumor tissues (r = 0.198, *P* = 0.008, Figure 4A). A significant association was also identified between the densities of IL-17+ TILs and CD57+ NK cells (r = 0.261, *P*<0.001, Figure 4B). From prognosis analysis, we found that higher levels of infiltrating CD8+ CTLs or CD57+NK cells correlated with a better overall survival of ESCC patients (*P*<0.001 and *P* = 0.002, respectively, determined by long-rank test, Figure 5).

## Discussion

Accumulating evidence suggests that IL-17 producing cells play a significant role in tumor immunity. However, IL-17 may play pro-tumor or anti-tumor effects in different tumor contexts [14], [18], [27], [28], [40]. Zhang et al found that IL-17 producing cells could promote tumor growth by stimulating angiogenesis in hepatocellular carcinoma patients [18]. Conversely, Kryczek et al demonstrated that IL-17 promoted antitumor activity in ovarian cancer patients [28]. The explanation for this discrepancy remains unknown.

We first detected tumor-infiltrating-IL-17 producing cells in human esophageal squamous cell carcinoma (ESCC) and observed that the levels of IL-17 producing cells correlated inversely with T (depth of primary tumor invasion, *P* = 0.042), indicating that enriched IL-17 producing cells in the tumor microenvironment may inhibit tumor invasion. Kaplan-Meier analysis revealed that increased levels of IL-17 producing cells were linked to better overall survival in ESCC patients (Figure 2, *P* = 0.031), indicating that IL-17 producing cell levels could potentially serve as a prognostic marker for ESCC. Our results are consistent with those of Kryczek et al, who demonstrated that the presence of IL-17+TILs correlated with favorable outcome in and enhanced survival of ovarian cancer patients [28]. Thus, in ESCC, our results suggest that IL-17 producing cells may mediate antitumor immunity.

We also detected CD8+ CTLs and CD57+ NK cells in tumor tissues from ESCC patients using serial tissue sections. Our study found that infiltrating CD8+ CTLs cells and CD57+ NK cells were present in ESCC tumors, and the abundance of CD8+ T or CD57+ cells correlated positively with the number of IL-17 producing cells. Martin-Orozco et al reported that Th17 cells can induce a strong antitumor CD8 response by eliciting the priming and recruitment of CD8+ T cells in a mouse model of lung melanoma [30]. Benchetrit et al found that IL-17 could inhibit tumor growth by inducing tumor-specific cytotoxic T lymphocyte (CTL) activity in hematopoietic tumors in immunocompetent mice [27]. In addition, Kryczek et al reported that both natural killer cell-mediated innate immunity and tumor-specific T-cell immunity were weakened in IL-17 deficient mice bearing MC38 tumors [29]; they also demonstrated that the abundance of IL-17 producing cells correlated positively with CD8+ T and NK cells in the same tumor microenvironment [28]. Our results are consistent with these studies, and indicate that IL-17 producing cells in ESCC might exert antitumor effects by enhancing cytotoxic T lymphocytes and NK cell responses.

In conclusion, our data show that IL-17 producing TILs were detected in ESCC, and the density of IL-17+ TILs was associated with better overall survival. Furthermore, the number of IL-17 producing cells correlated positively with the numbers of CD8+ CTLs and CD57 + NK cells. Thus, tumor infiltrating IL-17 producing cells may constitute a novel prognosis marker for ESCC and may play an antitumor role by activating innate and adaptive immunity.

## Materials and Methods

### Patients and tissue samples

Paraffin-embedded samples were obtained from 181 ESCC patients who underwent surgery at the Sun Yat-sen University Cancer Center between 2002 and 2003. There were 141 male and 40 female patients with a median age of 56 years (range, 33–79 years). Patients with autoimmune diseases and other esophageal cancers (e.g., adenocarcinoma) were excluded. None of the patients had received anticancer treatment prior to surgery. The follow-up data from the ESCC patients in this study are available and complete. There were 117 cases of stage I–II and 64 cases of stage III–IV cancer according to the American Joint Committee on Cancer (AJCC, 2002) TNM staging system. Each lesion was graded histologically according to the WHO classification criteria. Overall survival (OS) was defined as the interval between the date of surgery and date of death or the last known follow-up. The study was approved by the Ethics Committee of Sun Yat-sen University Cancer Center, and informed consent was obtained from each patient.

### Immunohistochemistry and immunofiuorescence

Formalin-fixed, paraffin-embedded samples were cut at a thickness of 2 µm. Each tissue section was deparaffinized and rehydrated through graded ethanol. For antigen retrieval, the slides were boiled in EDTA (1 mM, pH 8.0) for 15 min in a microwave oven. Endogenous peroxidase activity was blocked with 0.3% hydrogen peroxide solution for 10 min at room temperature. After rinsing with PBS, the slides were incubated overnight at 4°C with primary monoclonal antibodies, including goat anti-human IL-17 (R&D systems; dilution 1/300), mouse anti-CD8 (Zhong shan Golden Bridge Biotech., Beijing, China; dilution 1/100), rabbit anti-CD57 (Zhongshan Golden Bridge Biotech., Beijing, China; dilution 1/100). After three washes in PBS, sections were incubated with biotinylated secondary antibody (Zhongshan Golden Bridge Biotech., Beijing, China) for 30 min at room temperature. Finally, the visualization signal was developed with 3, 3′-diaminobenzidine tetrahydrochloride (DAB), and all slides were counterstained with hematoxylin. Some paraffin-embedded specimens were simultaneously incubated with goat anti-IL-17 (R&D systems; dilution 1/100) and rabbit anti-CD4 (Zhongshan Golden Bridge Biotech., Beijing, China; dilution 1/50), or with goat anti-IL-17 (R&D systems; dilution 1/100) and mouse anti-FOXP3 (abcam; dilution 1/100), followed by Phycoerythrin-conjugated Affinipure donkey anti-goat IgG(H+L) (Proteintech Group; dilution 1/50) and Fluorescein (FITC)-conjugated Affinipure donkey anti-rabbit IgG(H+L) (Proteintech Group; dilution 1/50), or by Alexa Fluor 488 donkey anti-goat IgG (H+L) (Molecular Probes; concentration 10 μg/ml) and Alexa Fluor 594 donkey anti-mouse IgG (H+L) (Molecular Probes; concentration 10 μg/ml).

Data were obtained by manually counting positively stained cells in ten separate fields under 400× high power magnification. The density of stained cells was determined by computing the mean number of positively stained cells per high power microscopic field (HPF).

### Statistical analysis

Quantitative values were expressed as means ± SD or median (range). Patients were divided into two groups based on the median of various immunohistochemical variables in our data (high level group vs. low level group). The Chi-square test or Fisher exact test was used to assess the relationships between the levels of IL-17 producing cells and clinicopathological features. The overall survival curves were calculated using the Kaplan-Meier method and analyzed using the long-rank test. Prognostic factors were examined by univariate and multivariate analyses using the Cox proportional hazards model. The correlation between the density of IL-17 producing cells and CD8+ CTLs or CD57+ NK cells were determined by Pearson correlation coefficient and linear regression analyses. A two-tailed *P*-value<0.05 was considered statistically significant. All statistical analyses were performed with SPSS software (version 16.0; SPSS Inc., Chicago, IL, USA).
